# LabEmbryoCam: An opensource phenotyping system for developing aquatic animals

**DOI:** 10.1016/j.ohx.2024.e00602

**Published:** 2024-11-09

**Authors:** Ziad Ibbini, Maria Bruning, Sakina Allili, Luke A Holmes, Ellen Tully, Jamie McCoy, Benjamin Larsen, Tony Wilson, Guy Ludford, Jack Barrett-Kelly, John I. Spicer, Oliver Tills

**Affiliations:** a University of Plymouth, School of Biological and Marine Sciences, Drake Circus, Plymouth, PL4 8AA, England; b Advanced Digital Manufacturing and Innovation Centre, Plymouth Science Park, 1 Davy Road, Plymouth, PL6 8BX, England

**Keywords:** High-dimensional-organismal-phenotyping, Phenomics, Computer vision, Embryos, Timelapse

## Abstract

Phenomics is the acquisition of high-dimensional data on an individual-wide scale and is proving transformational in areas of biological research related to human health including medicine and the crop sciences. However, more broadly, a lack of accessible transferrable technologies and research approaches is significantly hindering the uptake of phenomics, in contrast to molecular-omics for which transferrable technologies have been a significant enabler. Aquatic embryos are natural models for phenomics, due to their small size, taxonomic diversity, ecological relevance, and high levels of temporal, spatial and functional change. Here, we present LabEmbryoCam, an autonomous phenotyping platform for timelapse imaging of developing aquatic embryos cultured in a multiwell plate format, and while optimised for embryos, the instrument is extremely versatile. The LabEmbryoCam capitalises on 3D printing, single board computers, consumer electronics and stepper motor enabled motion. We combine these into a compact and modular laboratory insturment to provide X, Y and Z motion of a camera and lens, a web application streamlined for rapid setup of experiments, user email notifications and a humidification chamber to reduce evaporation over prolonged acquisitions. Downstream analyses are provided, enabling automated embryo segmentation, heartrate measurement, motion tracking, and energy proxy trait (EPT) measurement. The LabEmbryoCam is a scalable, and flexible laboratory instrument, that leverages embryonic and early life stage organisms to tackle key global challenges including biological sensitivity assessment, toxicological screening, but also to support broader engagement with the earliest stages of life.

## Graphical abstract

**Figure:**
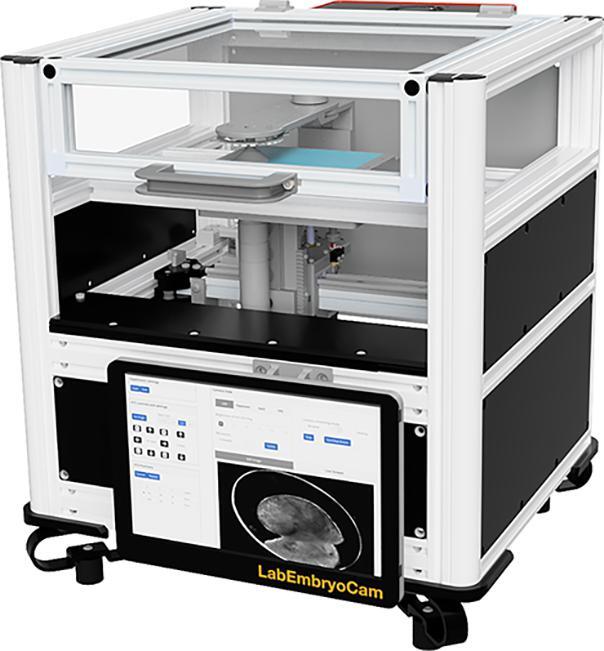

Specifications table.

**Table:** 

Hardware name	*LabEmbryoCam*
Subject area	Biological sciences
Hardware type	Imaging tools
Closest commercial analog	*Zantiks MWP**https://zantiks.com/products/zantiks-mwp**, nearest non-commercial analogue was the OpenVIM (Tills et al 2013 Proc Roy Soc B 280:20131479), but this was both high cost and required specialist engineering services.*
Open source license	*CERN OHL-P*
Cost of hardware	*£1350*
Source file repository	*If you’ve uploaded your source files to an approved repository (**OSF**,**Mendeley Data**or**Zenodo*) write the *DOI URL here.*https://doi.org/10.5281/zenodo.7575248

## 1 Hardware in context

LabEmbryoCam (LEC) is a comparatively low-cost laboratory phenotyping platform for the automated long-term imaging of developing aquatic embryos. The LEC provides a solution to the challenge of phenomics – high dimensional phenotyping of organisms [1], [2]. A lack of readily available and transferrable tools for phenomics is recognised as a key bottleneck to the advancement of biological research [3]. Aquatic embryos have emerged as natural models for phenomics. Embryos are scalable in both size and taxonomic diversity, and are also the most dynamic stage of life, often associated with heightened environmental sensitivity [4]. Automated timelapse imaging of aquatic embryos has proven a powerful method for assessing long term changes in growth and movement, alongside real-time physiological responses such as heart rate, behaviour and proxy traits [5], [6], [7], [8], [9], [10]. The LEC integrates custom open source hardware and software, optimised for the efficient and repeated imaging of embryos, over the course of days, weeks or even months in a multiwell plate format, for the duration of development, or an experiment. Templates are provided for efficient automated setup of common multiwell plate formats (24, 48, 96 and 384).

Minimising cost while also maximising versatility were key considerations of both the hardware and software of the LEC, to support its wider adoption across disciplines and geographical scales. While phenomics is becoming increasingly commonplace in areas of research closely linked to human health, particularly crop sciences and medicine, it remains challenging to apply phenomics more broadly in biological research, outside of particular model species such as zebrafish, for which some instruments do exist. *Daniovision*© and Zantiks MWP are commercial instruments for tracking small organisms within a multiwell plate format, with some support for assisted measurement of other traits such as heart rate. In contrast, the LEC was created to meet a specific research requirement – high-throughput, long term imaging of developing aquatic embryos, and is both comparatively low cost, and open-source. The establishment of transferrable tools and approaches is central to advancing the application of phenomics. The LEC capitalises on 3D printing, single-board computers (SBCs), low cost electronics and open-source software to offer a solution to the challenge of phenomics of organisms cultured in a multiwell plate format. Unlike molecular-omics, for which one instrument can be applicable to a broad range of biological systems and research questions, it is well acknowledged that solutions for phenomics are likely to require greater degrees of customization. Open source hardware and software should therefore be a priority to support the adoption and application of phenomics in research. We present the LEC as what we hope will be an accessible foundation from which variations may emerge to address a range of research challenges, and to capitalise on increased accessibility of 3D printing, embedded computing and computer-vision enabled approaches to image analysis Table 1.

**Table 1:** Design files for the hardware components of the LabEmbryoCam.

**Design file name**	**File type**	**Open source license**	**Location of the file**
LabEmbryoCam CAD model	*f3d*	*CERN-OHL-P*	https://doi.org/10.5281/zenodo.13328175
LEC_000_P_01_ELEC-MOUNT-TOP	*stl*	*CERN-OHL-P*	https://doi.org/10.5281/zenodo.13328175
LEC_000_P_02_ELEC-MOUNT-BOTTOM	*stl*	*CERN-OHL-P*	https://doi.org/10.5281/zenodo.13328175
LEC_001_P_01_DOOR-STOP-PLATE	*stl*	*CERN-OHL-P*	https://doi.org/10.5281/zenodo.13328175
LEC_002_P_03_LEAFSPRING	*stl*	*CERN-OHL-P*	https://doi.org/10.5281/zenodo.13328175
LEC_002_P_01_FR-BL_BOTTOMCORNER	*stl*	*CERN-OHL-P*	https://doi.org/10.5281/zenodo.13328175
LEC_002_P_02_FL-BR_BOTTOMCORNER	*stl*	*CERN-OHL-P*	https://doi.org/10.5281/zenodo.13328175
LEC_003_P_01_BACK-CORNER-L	*stl*	*CERN-OHL-P*	https://doi.org/10.5281/zenodo.13328175
LEC_003_P_02_BACK-CORNER-R	*stl*	*CERN-OHL-P*	https://doi.org/10.5281/zenodo.13328175
LEC_003_P_03_PULLEY-L	*stl*	*CERN-OHL-P*	https://doi.org/10.5281/zenodo.13328175
LEC_003_P_04_PULLEY-R	*stl*	*CERN-OHL-P*	https://doi.org/10.5281/zenodo.13328175
LEC_003_P_05_KNOB	*stl*	*CERN-OHL-P*	https://doi.org/10.5281/zenodo.13328175
LEC_004_P_04_TOP-CORNER-RIGHT	*stl*	*CERN-OHL-P*	https://doi.org/10.5281/zenodo.13328175
LEC_004_P_03_TOP-CORNER-LEFT	*stl*	*CERN-OHL-P*	https://doi.org/10.5281/zenodo.13328175
LEC_004_P_01_BOTTOM-CORNER-LEFT	*stl*	*CERN-OHL-P*	https://doi.org/10.5281/zenodo.13328175
LEC_004_P_02_BOTTOM-CORNER-RIGHT	*stl*	*CERN-OHL-P*	https://doi.org/10.5281/zenodo.13328175
LEC_004_P_05_LEFT-LOW-CORNER-BRACKET	*stl*	*CERN-OHL-P*	https://doi.org/10.5281/zenodo.13328175
LEC_005_P_03_Y-AXIS-UPPER-LEFT	*stl*	*CERN-OHL-P*	https://doi.org/10.5281/zenodo.13328175
LEC_005_P_04_Y-AXIS-UPPER-RIGHT	*stl*	*CERN-OHL-P*	https://doi.org/10.5281/zenodo.13328175
LEC_005_P_05_YAXIS-LIMTRIG	*stl*	*CERN-OHL-P*	https://doi.org/10.5281/zenodo.13328175
LEC_005_P_06_Y-AXIS-LIMSWITCH-MOUNT	*stl*	*CERN-OHL-P*	https://doi.org/10.5281/zenodo.13328175
LEC_005_P_01_YAXIS-LOWER	*stl*	*CERN-OHL-P*	https://doi.org/10.5281/zenodo.13328175
LEC_006_P_06-CARRIAGE-ROD-MOUNT	*stl*	*CERN-OHL-P*	https://doi.org/10.5281/zenodo.13328175
LEC_006_P_03_R-ROD-HOLDER	*stl*	*CERN-OHL-P*	https://doi.org/10.5281/zenodo.13328175
LEC_006_P_02_L-ROD-HOLDER	*stl*	*CERN-OHL-P*	https://doi.org/10.5281/zenodo.13328175
LEC_006_P_01_X-AXIS-CARRIAGE	*stl*	*CERN-OHL-P*	https://doi.org/10.5281/zenodo.13328175
LEC_006_P_04-L-ROD-STOP	*stl*	*CERN-OHL-P*	https://doi.org/10.5281/zenodo.13328175
LEC_006_P_05-R-ROD-STOP	*stl*	*CERN-OHL-P*	https://doi.org/10.5281/zenodo.13328175
LEC_006_A_X-CARRIAGE	*stl*	*CERN-OHL-P*	https://doi.org/10.5281/zenodo.13328175
LEC_007_P_09_BACK-ROD-SECURE	*stl*	*CERN-OHL-P*	https://doi.org/10.5281/zenodo.13328175
LEC_007_P_10_FRONT-ROD-SECURE	*stl*	*CERN-OHL-P*	https://doi.org/10.5281/zenodo.13328175
LEC_007_P_08_Z-LIM-TRIG	*stl*	*CERN-OHL-P*	https://doi.org/10.5281/zenodo.13328175
LEC_007_P_01_Z-CARRIAGE	*stl*	*CERN-OHL-P*	https://doi.org/10.5281/zenodo.13328175
LEC_007_P_06_RAIL_INSERT	*stl*	*CERN-OHL-P*	https://doi.org/10.5281/zenodo.13328175
LEC_007_P_11_LIGHT-MOUNT	*stl*	*CERN-OHL-P*	https://doi.org/10.5281/zenodo.13328175
LEC_007_P_15_OPTICS_MOUNT	*stl*	*CERN-OHL-P*	https://doi.org/10.5281/zenodo.13328175
LEC_007_P_02_LIGHT-BRACKET-TOP	*stl*	*CERN-OHL-P*	https://doi.org/10.5281/zenodo.13328175
LEC_007_P_03_LIGHT-BRACKET-BOTTOM	*stl*	*CERN-OHL-P*	https://doi.org/10.5281/zenodo.13328175
LEC_007_P_12_LIGHT-KNOB	*stl*	*CERN-OHL-P*	https://doi.org/10.5281/zenodo.13328175
LEC_007_P_13_LIGHT-DIFFUSER	*stl*	*CERN-OHL-P*	https://doi.org/10.5281/zenodo.13328175
LEC_007_P_17_PINION	*stl*	*CERN-OHL-P*	https://doi.org/10.5281/zenodo.13328175
LEC_007_P_16_RACK	*stl*	*CERN-OHL-P*	https://doi.org/10.5281/zenodo.13328175
LEC_007_P_14_LIGHT-SLIDER	*stl*	*CERN-OHL-P*	https://doi.org/10.5281/zenodo.13328175
LEC_007_P_18_3MM-STANDOFF	*stl*	*CERN-OHL-P*	https://doi.org/10.5281/zenodo.13328175
LEC_008_P_06_HDMI-CSI-MOUNT	*stl*	*CERN-OHL-P*	https://doi.org/10.5281/zenodo.13328175
LEC_008_P_07_FAN-PLATE	*stl*	*CERN-OHL-P*	https://doi.org/10.5281/zenodo.13328175
LEC_008_P_08_PORT-PLATE	*stl*	*CERN-OHL-P*	https://doi.org/10.5281/zenodo.13328175
LEC_008_P_01_MOUNTING-PLATE	*stl*	*CERN-OHL-P*	https://doi.org/10.5281/zenodo.13328175
LEC_008_P_05_MICROCONTROLLER-BRACKET	*stl*	*CERN-OHL-P*	https://doi.org/10.5281/zenodo.13328175
LEC_009_P_04_DISPLAY-FASCIA	*stl*	*CERN-OHL-P*	https://doi.org/10.5281/zenodo.13328175
LEC_009_P_03_DISPLAY-BRACKET	*stl*	*CERN-OHL-P*	https://doi.org/10.5281/zenodo.13328175
LEC_009_P_02_DISPLAY-ARM	*stl*	*CERN-OHL-P*	https://doi.org/10.5281/zenodo.13328175
LEC_009_P_06_DISPLAY-KNOB	*stl*	*CERN-OHL-P*	https://doi.org/10.5281/zenodo.13328175
LEC_009_P_05_DISPLAY-REAR	*stl*	*CERN-OHL-P*	https://doi.org/10.5281/zenodo.13328175
LEC_009_P_01_DISPLAY-BRACKET	*stl*	*CERN-OHL-P*	https://doi.org/10.5281/zenodo.13328175
LEC_011_P_01_L-FRONT-PANEL	*stl*	*CERN-OHL-P*	https://doi.org/10.5281/zenodo.13328175
LEC_011_P_02_R-FRONT-PANEL	*stl*	*CERN-OHL-P*	https://doi.org/10.5281/zenodo.13328175
LEC_012_P_01_HUMIDIFICATION-CHAMBER	*stl*	*CERN-OHL-P*	https://doi.org/10.5281/zenodo.13328175
LEC_012_P_02_HUMIDIFICATION-CHAMBER-LID	*stl*	*CERN-OHL-P*	https://doi.org/10.5281/zenodo.13328175
LEC_012_P_01_HUMIDIFICATION-CHAMBER-GLASS-SECURE	*stl*	*CERN-OHL-P*	https://doi.org/10.5281/zenodo.13328175
LEC_012_P_01_DRESCHEL-BOTTLE-SECURE	*stl*	*CERN-OHL-P*	https://doi.org/10.5281/zenodo.13328175
LEC_012_P_01_DRESCHEL-BOTTLE-STAND	*stl*	*CERN-OHL-P*	https://doi.org/10.5281/zenodo.13328175

## 2 Hardware description

All design files are accessible at https://doi.org/10.5281/zenodo.7575248.

**Fig. 1:**
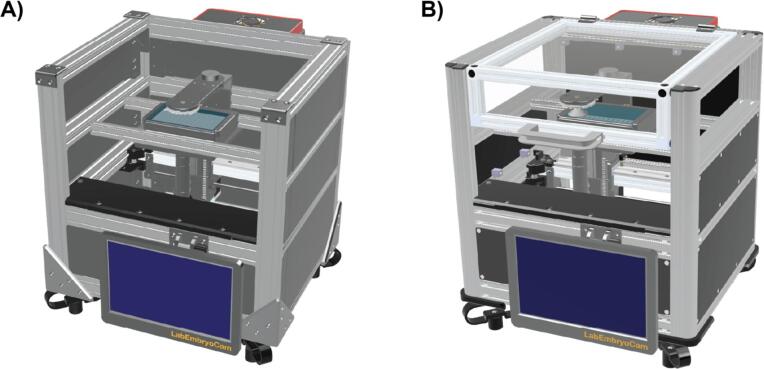
CAD rendering of assembled different versions of the LabEmbryoCam (LEC). A) V1 LEC is built using generic aluminium extrusion and self-supporting brackets, and B) V2 LEC is built using Rexroth aluminium extrusion, blind joints and includes a lid. Dimensions: 450 mm wide, 390 mm deep, 475 mm tall.

The LabEmbryoCam (LEC) is a laboratory imaging instrument built within an aluminium extrusion framework, upon which FDM 3D printed parts are mounted (Fig. 1). X and Y motion of a Z-carriage holding the imaging apparatus is provided via stepper motors driving a CoreXY belt drive setup. The Z-carriage contains a lens, camera and LED ring light, designed for dark field lighting, and a stepper motor and leadscrew-driven linear actuator for vertical movement of the optics in the vertical (Z) direction to provide focus capability. Where practical, 3D printed parts are used, to improve opportunity for innovation, minimising cost and limiting reliance on supply chains. The LEC is highly modular, enabling use of different imaging components, or modification of the design to operate at different scales or resolutions. The instrument is stand-alone with a custom user interface for hardware control and experimental setup, accessed via a touchscreen mounted to the front of instrument. The LEC presented here uses low-cost optics inverted beneath a multiwell plate, including a Raspberry Pi HQ 12 MP camera, or Raspberry Pi Global Shutter Camera, and low-cost microscope lens (0.12–1.8 x magnification), with a combined optics cost of approximately GBP £100.

Electronics are mounted on the rear of the instrument, within an electronics enclosure, to aid assembly, provide some environmental protection and to simplify setup. Electronic control systems comprise a PC power supply, an Arduino UNO microcontroller, for controlling the lighting, a Duet 2 microcontroller for XYZ stepper motor control, a powered USB hub, an ATX power supply splitter, a Raspberry Pi 4 model B SBC and an SSD for data storage.

A key challenge during timelapse imaging of aquatic animals in multiwell plate formats is evaporation, and where this occurs it causes distorted lighting of embryos and altered water chemistry. Here, we provide a solution to this challenge in the form of a 3D printed humidification chamber, enabling long term autonomous imaging of developing aquatic embryos with limited maintenance.

Commercially available solutions for insulation against environmental vibration are costly. Here, we provide a low cost yet effective solution to reducing the impact of environmental vibration during imaging – integrating a Sorbothane foot, and a 3D printed leafspring mounted on each corner.

User control of the LEC is achieved through a Python web application, on a touch screen mounted to the front of the instrument. While the interface will work on any display connected to the electronics enclosure via HDMI, a touchscreen is incorporated into the design to make the instrument standalone. The user interface enables efficient setup of long-term multi-dimensional image acquisitions, integrating repeated video acquisition of individual embryos over prolonged periods, with autonomous control of the camera, XYZ motion and lighting. Functionality for user updates of an experiment's progress, via email is provided for remote monitoring, and owing to the use of a Dash server-based user interface there is also capability for accessing instruments via a local area network (LAN), as opposed to touch screen, or direct monitor, keyboard and mouse connections, if preferred.

Key features of the LabEmbryoCam include:

• A significant range of movement (130 mm x 85 mm x 50 mm), with a minimal incremental movement of 10 µm, highly appropriate to multiwell plate formats.
• Optimised software for efficient acquisition of video at high frame rates and resolutions, using a Raspberry Pi 4 model B.
• A capability for stable acquisition of timelapse video over prolonged periods to support downstream analytical processes for producing high-dimensional phenome level datasets.
• Integration of downstream processes including video compilation and image analysis pipelines, including egg localisation and embryo segmentation, optical flow motion analysis, heart rate quantification and machine proxy traits including energy proxy traits.
• Humidification chamber to reduce evaporation for stable long-term imaging of aquatic organisms cultured in a multiwell plate.
• Integrated vibration insulation to support long-term timelapse imaging.

## 3 Design files

### 3.1 CAD files

An Autodesk Fusion CAD file for the LabEmbryoCam is available from the paper DOI, and the CAD files are also embedded into the online build guide.

*3D printing.* The LabEmbryoCam incorporates > 100 3D printed parts. All STLs are available *via* the Zenodo repository accessible at https://doi.org/10.5281/zenodo.7575248, alongside suggested print settings and part orientations.

*Electronics:* No custom PCBs are used in this instrument. Fig. 10 outlines the connection of the various electronic components to one another, and this is also made clear in both the Python and microcontroller scripts.

### 3.2 Software and firmware

The LabEmbryoCam is controlled via a web application, built with Python using Dash, running on a Raspberry Pi 4 model B (SBC), connected to an Arduino UNO microcontroller for controlling LED brightness, and a Duet 2 for XYZ stepper motor control. The firmware for each microcontroller is provided in the project Github repository.

A disk image is provided for the Raspberry Pi with all the necessary prerequisite packages preinstalled.

**Table:** 

**Design file name**	**File type**	**Open source license**	**Location of the file**
LabEmbryoCam CAD model	*f3d*	*CERN-OHL-P*	https://doi.org/10.5281/zenodo.7575248
LEC_000_P_01_ELEC-MOUNT-TOP	*stl*	*CERN-OHL-P*	https://doi.org/10.5281/zenodo.7575248
LEC_000_P_02_ELEC-MOUNT-BOTTOM	*stl*	*CERN-OHL-P*	https://doi.org/10.5281/zenodo.7575248
LEC_001_P_01_DOOR-STOP-PLATE	*stl*	*CERN-OHL-P*	https://doi.org/10.5281/zenodo.7575248
LEC_002_P_03_LEAFSPRING	*stl*	*CERN-OHL-P*	https://doi.org/10.5281/zenodo.7575248
LEC_002_P_01_FR-BL_BOTTOMCORNER	*stl*	*CERN-OHL-P*	https://doi.org/10.5281/zenodo.7575248
LEC_002_P_02_FL-BR_BOTTOMCORNER	*stl*	*CERN-OHL-P*	https://doi.org/10.5281/zenodo.7575248
LEC_003_P_01_BACK-CORNER-L	*stl*	*CERN-OHL-P*	https://doi.org/10.5281/zenodo.7575248
LEC_003_P_02_BACK-CORNER-R	*stl*	*CERN-OHL-P*	https://doi.org/10.5281/zenodo.7575248
LEC_003_P_03_PULLEY-L	*stl*	*CERN-OHL-P*	https://doi.org/10.5281/zenodo.7575248
LEC_003_P_04_PULLEY-R	*stl*	*CERN-OHL-P*	https://doi.org/10.5281/zenodo.7575248
LEC_003_P_05_KNOB	*stl*	*CERN-OHL-P*	https://doi.org/10.5281/zenodo.7575248
LEC_004_P_04_TOP-CORNER-RIGHT	*stl*	*CERN-OHL-P*	https://doi.org/10.5281/zenodo.7575248
LEC_004_P_03_TOP-CORNER-LEFT	*stl*	*CERN-OHL-P*	https://doi.org/10.5281/zenodo.7575248
LEC_004_P_01_BOTTOM-CORNER-LEFT	*stl*	*CERN-OHL-P*	https://doi.org/10.5281/zenodo.7575248
LEC_004_P_02_BOTTOM-CORNER-RIGHT	*stl*	*CERN-OHL-P*	https://doi.org/10.5281/zenodo.7575248
LEC_004_P_05_LEFT-LOW-CORNER-BRACKET	*stl*	*CERN-OHL-P*	https://doi.org/10.5281/zenodo.7575248
LEC_005_P_03_Y-AXIS-UPPER-LEFT	*stl*	*CERN-OHL-P*	https://doi.org/10.5281/zenodo.7575248
LEC_005_P_04_Y-AXIS-UPPER-RIGHT	*stl*	*CERN-OHL-P*	https://doi.org/10.5281/zenodo.7575248
LEC_005_P_05_YAXIS-LIMTRIG	*stl*	*CERN-OHL-P*	https://doi.org/10.5281/zenodo.7575248
LEC_005_P_06_Y-AXIS-LIMSWITCH-MOUNT	*stl*	*CERN-OHL-P*	https://doi.org/10.5281/zenodo.7575248
LEC_005_P_01_YAXIS-LOWER	*stl*	*CERN-OHL-P*	https://doi.org/10.5281/zenodo.7575248
LEC_006_P_06-CARRIAGE-ROD-MOUNT	*stl*	*CERN-OHL-P*	https://doi.org/10.5281/zenodo.7575248
LEC_006_P_03_R-ROD-HOLDER	*stl*	*CERN-OHL-P*	https://doi.org/10.5281/zenodo.7575248
LEC_006_P_02_L-ROD-HOLDER	*stl*	*CERN-OHL-P*	https://doi.org/10.5281/zenodo.7575248
LEC_006_P_01_X-AXIS-CARRIAGE	*stl*	*CERN-OHL-P*	https://doi.org/10.5281/zenodo.7575248
LEC_006_P_04-L-ROD-STOP	*stl*	*CERN-OHL-P*	https://doi.org/10.5281/zenodo.7575248
LEC_006_P_05-R-ROD-STOP	*stl*	*CERN-OHL-P*	https://doi.org/10.5281/zenodo.7575248
LEC_006_A_X-CARRIAGE	*stl*	*CERN-OHL-P*	https://doi.org/10.5281/zenodo.7575248
LEC_007_P_09_BACK-ROD-SECURE	*stl*	*CERN-OHL-P*	https://doi.org/10.5281/zenodo.7575248
LEC_007_P_10_FRONT-ROD-SECURE	*stl*	*CERN-OHL-P*	https://doi.org/10.5281/zenodo.7575248
LEC_007_P_08_Z-LIM-TRIG	*stl*	*CERN-OHL-P*	https://doi.org/10.5281/zenodo.7575248
LEC_007_P_01_Z-CARRIAGE	*stl*	*CERN-OHL-P*	https://doi.org/10.5281/zenodo.7575248
LEC_007_P_06_RAIL_INSERT	*stl*	*CERN-OHL-P*	https://doi.org/10.5281/zenodo.7575248
LEC_007_P_11_LIGHT-MOUNT	*stl*	*CERN-OHL-P*	https://doi.org/10.5281/zenodo.7575248
LEC_007_P_15_OPTICS_MOUNT	*stl*	*CERN-OHL-P*	https://doi.org/10.5281/zenodo.7575248
LEC_007_P_02_LIGHT-BRACKET-TOP	*stl*	*CERN-OHL-P*	https://doi.org/10.5281/zenodo.7575248
LEC_007_P_03_LIGHT-BRACKET-BOTTOM	*stl*	*CERN-OHL-P*	https://doi.org/10.5281/zenodo.7575248
LEC_007_P_12_LIGHT-KNOB	*stl*	*CERN-OHL-P*	https://doi.org/10.5281/zenodo.7575248
LEC_007_P_13_LIGHT-DIFFUSER	*stl*	*CERN-OHL-P*	https://doi.org/10.5281/zenodo.7575248
LEC_007_P_17_PINION	*stl*	*CERN-OHL-P*	https://doi.org/10.5281/zenodo.7575248
LEC_007_P_16_RACK	*stl*	*CERN-OHL-P*	https://doi.org/10.5281/zenodo.7575248
LEC_007_P_14_LIGHT-SLIDER	*stl*	*CERN-OHL-P*	https://doi.org/10.5281/zenodo.7575248
LEC_007_P_18_3MM-STANDOFF	*stl*	*CERN-OHL-P*	https://doi.org/10.5281/zenodo.7575248
LEC_008_P_06_HDMI-CSI-MOUNT	*stl*	*CERN-OHL-P*	https://doi.org/10.5281/zenodo.7575248
LEC_008_P_07_FAN-PLATE	*stl*	*CERN-OHL-P*	https://doi.org/10.5281/zenodo.7575248
LEC_008_P_08_PORT-PLATE	*stl*	*CERN-OHL-P*	https://doi.org/10.5281/zenodo.7575248
LEC_008_P_01_MOUNTING-PLATE	*stl*	*CERN-OHL-P*	https://doi.org/10.5281/zenodo.7575248
LEC_008_P_05_MICROCONTROLLER-BRACKET	*stl*	*CERN-OHL-P*	https://doi.org/10.5281/zenodo.7575248
LEC_009_P_04_DISPLAY-FASCIA	*stl*	*CERN-OHL-P*	https://doi.org/10.5281/zenodo.7575248
LEC_009_P_03_DISPLAY-BRACKET	*stl*	*CERN-OHL-P*	https://doi.org/10.5281/zenodo.7575248
LEC_009_P_02_DISPLAY-ARM	*stl*	*CERN-OHL-P*	https://doi.org/10.5281/zenodo.7575248
LEC_009_P_06_DISPLAY-KNOB	*stl*	*CERN-OHL-P*	https://doi.org/10.5281/zenodo.7575248
LEC_009_P_05_DISPLAY-REAR	*stl*	*CERN-OHL-P*	https://doi.org/10.5281/zenodo.7575248
LEC_009_P_01_DISPLAY-BRACKET	*stl*	*CERN-OHL-P*	https://doi.org/10.5281/zenodo.7575248
LEC_011_P_01_L-FRONT-PANEL	*stl*	*CERN-OHL-P*	https://doi.org/10.5281/zenodo.7575248
LEC_011_P_02_R-FRONT-PANEL	*stl*	*CERN-OHL-P*	https://doi.org/10.5281/zenodo.7575248
LEC_012_P_01_HUMIDIFICATION-CHAMBER	*stl*	*CERN-OHL-P*	https://doi.org/10.5281/zenodo.7575248
LEC_012_P_02_HUMIDIFICATION-CHAMBER-LID	*stl*	*CERN-OHL-P*	https://doi.org/10.5281/zenodo.7575248
LEC_012_P_01_HUMIDIFICATION-CHAMBER-GLASS-SECURE	*stl*	*CERN-OHL-P*	https://doi.org/10.5281/zenodo.7575248
LEC_012_P_01_DRESCHEL-BOTTLE-SECURE	*stl*	*CERN-OHL-P*	https://doi.org/10.5281/zenodo.7575248
LEC_012_P_01_DRESCHEL-BOTTLE-STAND	*stl*	*CERN-OHL-P*	https://doi.org/10.5281/zenodo.7575248

The location of all STLs are detailed in the CAD file, and referred to in the build guide both in this paper and at https://labembryocam.readthedocs.io/en/latest/.

## 4 Bill of materials

The LabEmbryoCam BOM includes > 550 fixings, >100 3D printed parts, mechanical and electronic parts. It is accessible as a spreadsheet at https://doi.org/10.5281/zenodo.7575248, providing direct links to product pages, costs, and model numbers, but prices may vary. CAD interactive views of the various subassemblies are also accessible at www.phenomyx.co.uk. Links in the BOM are provided to limit the number of individual orders, and associated shipping charges, but parts will be available from a range of suppliers. Fully assembled instruments are now available for purchase directly from the originators of the instrument, with details accessible at www.phenomyx.co.uk.

The instrument built and tested here is using linear rails and carriages from Igus (Cologne, Germany), cheaper rails could be used, but they are chosen here to maximise reliability.

## 5 Build instructions

**Fig. 2:**
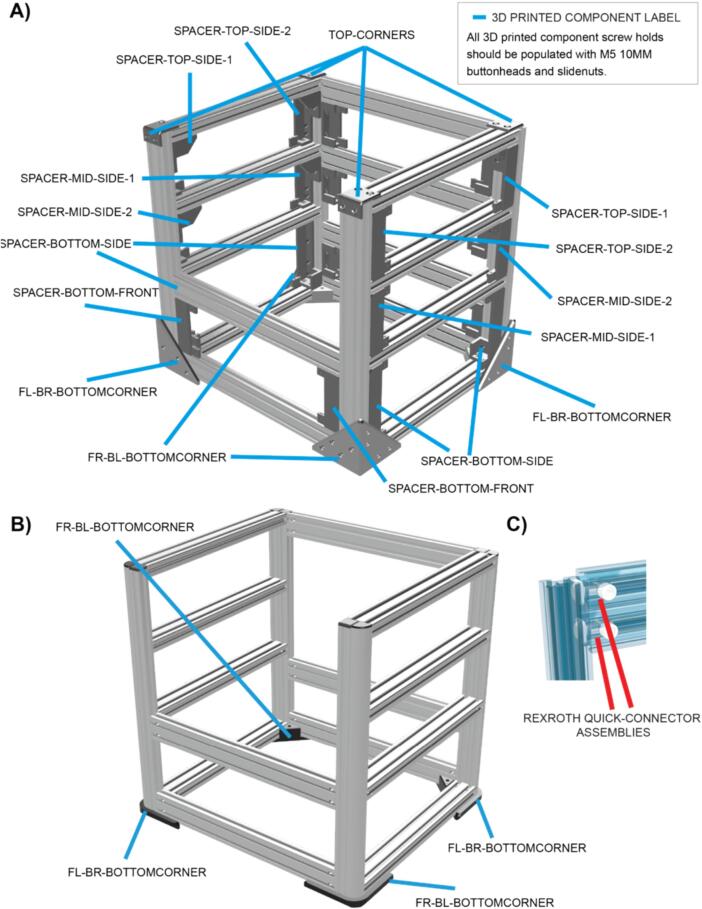
**A)** Assembly schematic for LEC V1 using generic aluminium extrusion. **B)** Assembly schematic for the LEC V2 using Bosch Rexroth aluminium extrusion. **C)** Rexroth quick-connector components for attaching frames.

The LabEmbryoCam is built using readily available and low-cost consumer electronics, mechanical parts and 3D printed parts. There is significant scope for changing components, or suppliers due to availability, price, or user requirements. To this end, we make available two versions of the LabEmbryoCam (Fig. 2).

- LabEmbryoCam V1 is built using generic aluminium extrusion – cut using a hacksaw, with no need for precision drilling, or tapping.
- LabEmbryoCam V2 is built using Bosch Rexroth aluminium extrusion, which can be bought as an assembled frame from suppliers of Rexroth, or individual lengths bought and cut. While the Bosch (Abstatt, Germany) Rexroth frame adds cost, it lowers the barrier to entry for laboratories without workshop facilities and it is also more robust.

Much of the assembly is the same for V1 and V2, however where appropriate, CAD and 3D print model files are labelled with either V1 or V2, according to which version of the instrument you are building. Furthermore, for the build instructions below, where the process differs between versions of the instrument this is made clear.

STLs are provided for all 3D printed parts, but so too are pre-sliced build plates optimized for printing the parts for one LabEmbryoCam. The instrument in this paper was produced using open-source Prusa (Prague, Czech Republic) MK3S 3D printers. One Prusa MK3S printer and construct all of the parts for one LabEmbryoCam in 10 prints, or approximately 500 h of print time. The type of plastic filament used for printing will generally not impact the structure of the main frame, but we use recycled Filamentive PETG filament (Filamentive, Sheffield, UK). Where possible, chopped carbon-fibre PETG (CF-PETG) is recommended for the Z-carriage stiffness and to reduce the weight of the z-carriage parts.

The LabEmbryoCam is modelled in Fusion360 and in addition to the model file, an online build guide (https://www.phenomyx.co.uk/) is available with embedded interactive 3D models to visualize the location of individual components.

An assembled LabEmbryoCam is 470 mm deep x 450 mm wide x 475 mm tall. The outer plastic panels and lid are optional, as they are not central to the function of the instrument, but do provide protection to the internal components and help to keep the instrument clean.

Sub-Assembly 1 – Frame.

The LabEmbryoCam hardware is modular, and versatile. Instructions are provided for building instruments using two different types of aluminium extrusion – generic extrusion, and Bosch Rexroth extrusion. While Bosch Rexroth extrusion is more expensive and less widely available, it is assembled using blind joints, requiring fewer 3D printed parts, and results in a more robust and refined instrument.

V1: Use the V1 version of the LEC_002_P_02_A_FL-BR-BOTTOMCORNER and LEC_002_P_02_A-FR-BL-BOTTOMCORNER as a basis to begin building the frame from the bottom layer, upwards. V1 frames are built using 3D printed spacer components to structure the frame.

V2: Fit the V2 version of LEC_002_P_02_A-FL-BR-BOTTOMCORNER and LEC_002_P_02_A-FR-BL-BOTTOMCORNER, to the front left/back right front right/back left corners respectively – using M5 12 MM BUTTONHEAD screws and M5 SLIDE-NUTS.

Sub-Assembly 2 – Rear Corners.

**Fig. 3:**
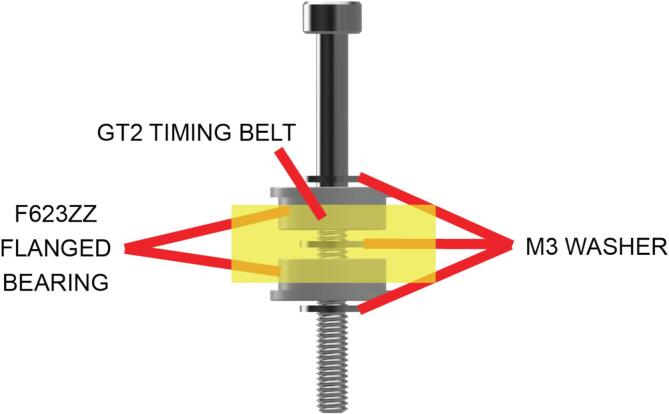
Pulley assembly for routing the X and Y axis timing belt.

X and Y motion in the LEC relies on a timing belt driven CoreXY system of motion. Pulleys are used to route the timing belts, and these pulleys are constructed using flanged bearings and washers, mounted in parts of the instrument in the front and rear corners, the y-axis and the x-axis (Fig. 3).

**Fig. 4:**
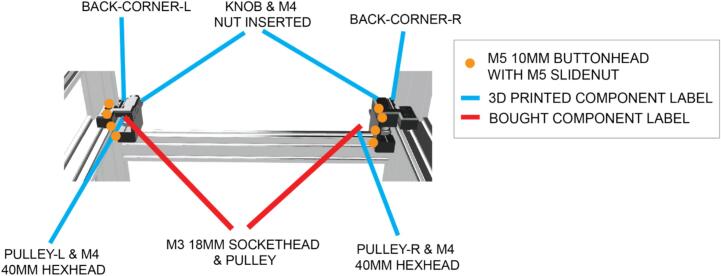
Rear corner assembly components, including 3D printed parts, fixings and bought components.

Each of the rear corners of the instrument consist of two 3D printed components, that route the X and Y timing belt (Fig. 4).

Sub-Assembly 3 – Front Corners.

**Fig. 5:**
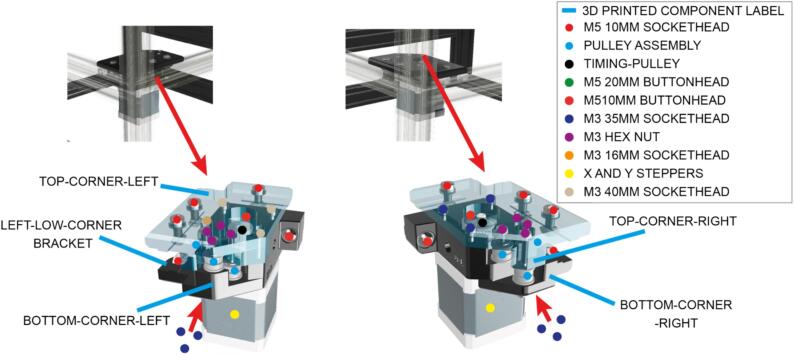
Fixings for left and right front corner assemblies.

Each front corner (Fig. 5) encompass three pulleys (Fig. 3), a stepper motor and fixings, to route the timing belts responsible for delivering X and Y motion, from the stepper motors in each front corner (Fig. 5). The left and right front corners are not symmetrical.

The lower parts of the front corners should be flush with the top of the lateral 2040 pieces of extrusion to which they are now attached. Begin by attaching these parts to the aluminium extrusion. Note the location of the different screw lengths in the front corners. Insert the M3 35 mm screws from underneath until they protrude sufficiently to install the parts required to mount the pulleys (Fig. 4).

Before attaching the top front corner part, it is important to first route the timing belts, taking care not to dislodge the components of the pulleys.

Sub-Assemblies 4 and 5 – X and Y-axes.

**Fig. 6:**
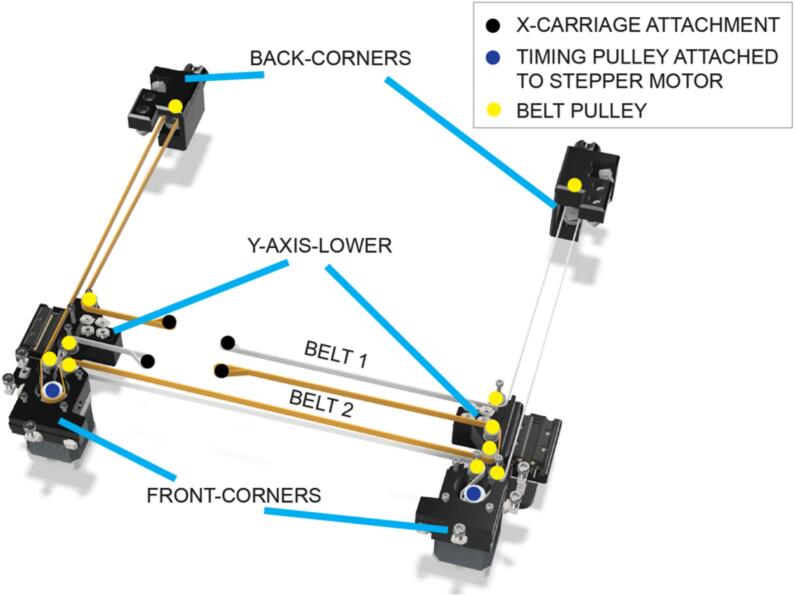
Routing of the X and Y motion timing belts around the pulleys to achieve CoreXY motion of the X-axis carriage.

**Fig. 7:**
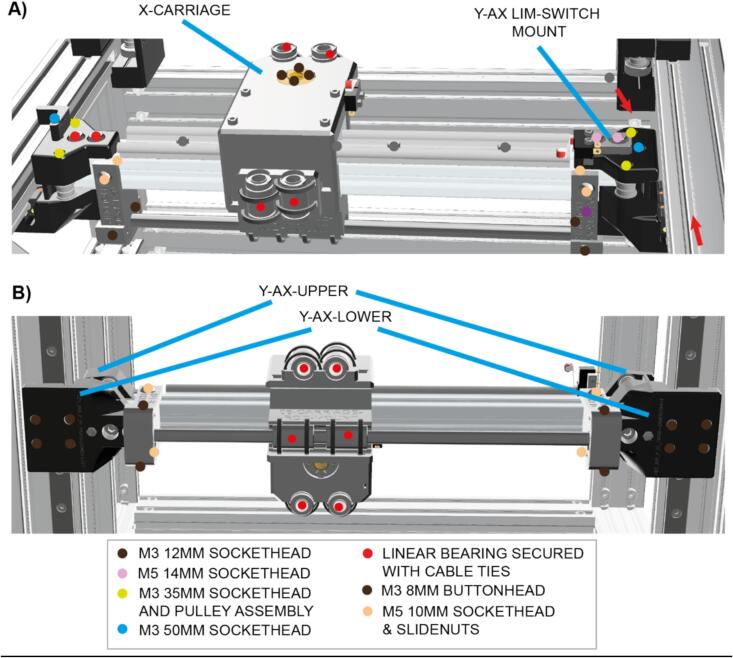
Annotated CAD render of the X-axis assembly.

X and Y motion is achieved using a CoreXY motion system (Fig. 6). The x-axis relies on a 320 mm piece of 2020 extrusion that moves front to back along the y-axis. It is easiest to build the x-axis outside of the instrument and then attach it to the carriages on the two y-axis linear rails that were previously installed (Fig. 7). Furthermore, each end of the X-axis incorporates two pulleys for routing the belts (the pulley assembly is detailed in Fig. 3).

Sub-Assembly 6 – Z-axis.

The z-axis incorporates the optics and lighting, but also the mechanism for z-axis motion. This all runs on the X-CARRIAGE (Fig. 7) from side to side on a linear rail, mounted on a 2020 piece of x-axis extrusion. The X-CARRIAGE is the first piece of this assembly to be installed onto the linear rail carriage. This part stays stationary in the z-direction and provides the base from which the z-assembly then moves up and down. Before attaching the X-CARRIAGE component to the linear rail carriage, install cable ties through each of the two holes on both the front and back of the X-CARRIAGE. This is harder to do once it is attached to the x-axis linear rail carriage.

**Fig. 8:**
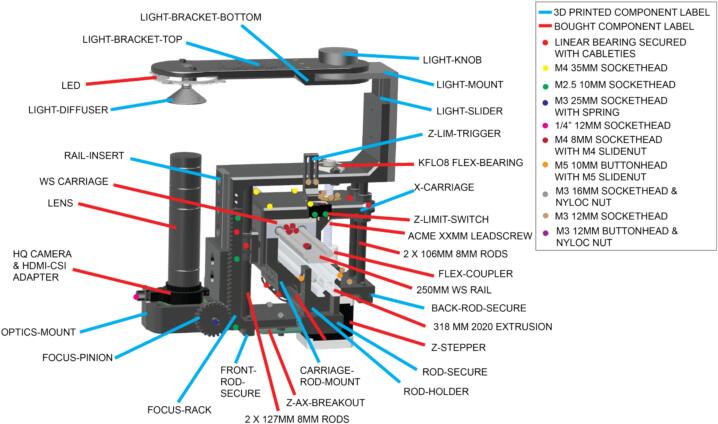
Annotated CAD render of the z-axis assembly.

The sequence of the Z-AXIS assembly steps is critical to prevent the need for subsequent disassembly (Fig. 8).

1. Slide the 2 x 127MM 8MM RODS at the front of the X-CARRIAGE through the LINEAR BEARINGS.

2. Attach the Z-STEPPER motor to the back of the Z-AXIS.

3. Attach the RAIL INSERT to the front of the Z-AXIS.

4. Insert 2 x 106MM ROD into the back of the X-CARRIAGE, with a linear bearing on each, but this must be done with the assembly attached to the linear rail. Cable ties can then be used to secure the linear bearings on both the front and the back rods, to the z-carriage. The leadscrew should then be fed in from the top down through the KFL08 bearing and leadscrew nut to the stepper motor coupler and secured with the grub screws on the coupler.

Sub-Assembly 7 – Feet.

**Fig. 9:**
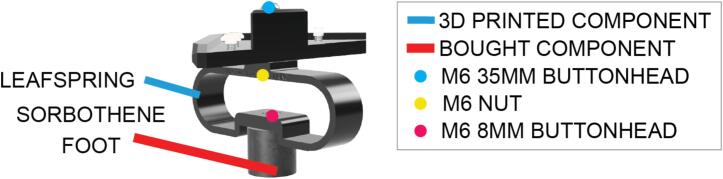
Annotated foot assembly to ensure vibration insulation during operation, integrating a 3D printed leaf spring for insulating lower frequencies, and sorbothane foot for insulating higher frequencies.

Feet insulate the instrument against vibration and consist of a 3D printed leafspring and a Sorbothane foot (Fig. 9).

Sub-Assembly 8 − Electronics.

**Fig. 10:**
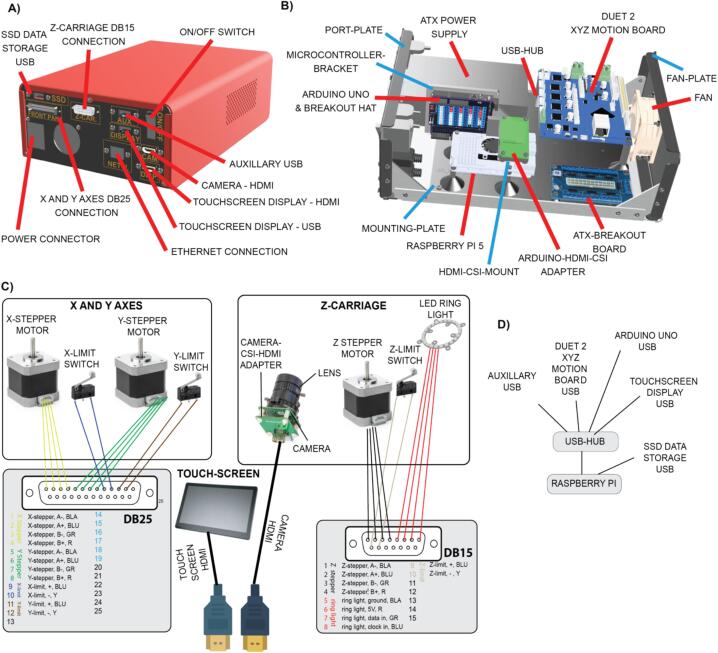
Electronics assembly **A)** Electronics enclosure port plate connections. **B)** Electronics enclosure assembly. **C)** Wiring schematic detailing the wiring pin outs from the electronics enclosure. **D)** Schematic of USB connections in the electronics enclosure.

The electronics are housed within the ELECTRONICS-ENCLOSURE, on a 3D printed MOUNTING-PLATE. Two face plates – the PORT-PLATE and the FAN-PLATE are mounted either end of the enclosure (Fig. 10).

### 5.1 Lighting microcontroller

The LED-MICROCONTROLLER should be programmed using the freely available Arduino IDE: https://support.arduino.cc/hc/en-us/articles/4733418441116-Upload-a-sketch-in-Arduino-IDE.

The LEC achieves darkfield lighting using a Sparkfun 2 inch 40 LED light ring, and a 3D printed LIGHT-DIFFUSER. The LED is driven *via* an Arduino UNO microcontroller, and light intensity is controllable via the LEC UI on the Raspberry Pi, with a relatively simple script. There is significant scope for different lighting solutions in the LEC, to suit different sample sites, or lenses.

### 5.2 XYZ motion controller

The LabEmbryoCam makes use of a CoreXY (https://en.wikipedia.org/wiki/CoreXY) style of motion control and this can be achieved using a range of different microcontroller boards from both CNC and 3D printing suppliers. The DUET 2 XYZ MOTION BOARD is used here due to the integration of quiet stepper motor drivers, with capacity for reduced power consumption when steppers are idle. However, other XYZ stepper motor boards could be used.

Firmware and configuration files for the Duet board are accessible in the XYZ_microcontroller folder of the LabEmbryoCam software directory, and this simply needs copying to the microSD card in the DUET2 XYZ MOTION BOARD.

### 5.3 Raspberry Pi

A Raspberry Pi 4 4 GB can run the LEC smoothly, but Raspberry Pi 5 is noticeably more responsive when using the user-interface. Nonetheless, the LEC software can be run from any computer on which the Raspberry Pi HQ or global shutter cameras and Dash are supported, including NVIDIA Jetson powered single board computers. Furthermore, the LEC hardware and software is versatile and can be modified to accommodate different camera models, and therefore can operate on different compute platforms. All testing here has been done using a Raspberry Pi 4 model B 8 GB.

A microSD for the Raspberry Pi can be flashed with a precompiled image containing the Bookworm OS and all LEC dependencies, but if preferred a microSD card can be prepared from scratch using the Raspberry Pi Imager software − https://www.raspberrypi.com/software, and installing the documented LEC dependencies. The Raspberry Pi is powered by the 5 V power supply from the ATX splitter, connected to a USB-C cable.

Sub-Assembly 9 – Humidification chamber.

**Fig. 11:**
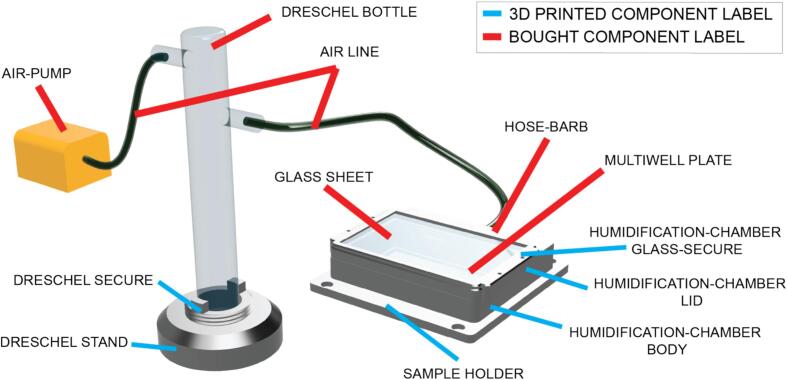
Annotated CAD render of the humidification embryo culture assembly.

Evaporation and condensation are common challenges for any long-term imaging of aquatic samples, but particularly small samples such as embryos. We have designed a simple solution to this challenge in the form of a 3D printed humidification chamber (Fig. 11). The humidification chamber connects to a Dreschel bottle containing water, connected to an air pump, to pass humidified air over a multiwell plate, thereby reducing evaporation and condensation. Testing has shown the humidification chamber achieves > 90 % humidity and reduces evaporation from individual wells of a multiwell plate to negligible levels. The humidification chamber is not essential to the use of the LabEmbryoCam, and 3D printable mounts for multiwell plates and Petri dishes are also provided.

### 5.4 Design considerations

Reduced cost, scalability and reliability were central considerations during the design of the LabEmbryoCam, and so too was insulating the instrument against vibration in the environment (Fig. 9). Inspiration is taken from CoreXY style 3D printers for the style of motion and the choice of readily available components. The LabEmbryoCam size could easily be scaled both up or down, by using different length linear rails and the according lengths of aluminium extrusion.

### 5.5 Safety Concerns

The LabEmbryoCam provides X, Y and Z movement and there is therefore the danger of entrapment of hands. This is limited by the use of aluminium extrusion to surround the instrument, with the recommendation to add acrylic panels. The use of belts for X and Y movement also results in stalling of the motion, or skipping of the belts over the timing pulleys, if significant resistance is encountered, such as is the case if an obstacle is met.

Electronics in the instrument are powered at 5 V, except for the XYZ stepper driver board, which requires a 12 V input. A computer power supply is used to provide power at these two voltages. Both the Raspberry Pi and XYZ stepper driver board should be fitted with heatsinks, and both are cooled from the ELECTRONICS-ENCLOSURE FAN. Care should be taken if the LabEmbryoCam is used in particularly wet environments. There is potential for extending the cables to the electronics enclosure to enable the electronics to be mounted in a more suitable environment.

## 6 Operation instructions

### 6.1 Setting LabEmbryoCam up

• Plug the LabEmbryoCam into the mains power supply, via a kettle cable.
• Follow the software installation instructions above and copy across the most recent version of the LEC software from Github.
• If an internet connection is available, either *via* ethernet, or wifi, the LabEmbryoCam can send notifications of the progress of an acquisition – enabling remote monitoring. Wifi can be setup using the Raspbian operating system. Note, however, that some organizational networks are not compatible with Raspberry Pi.

### 6.2 Launching user interface

**Fig. 12:**
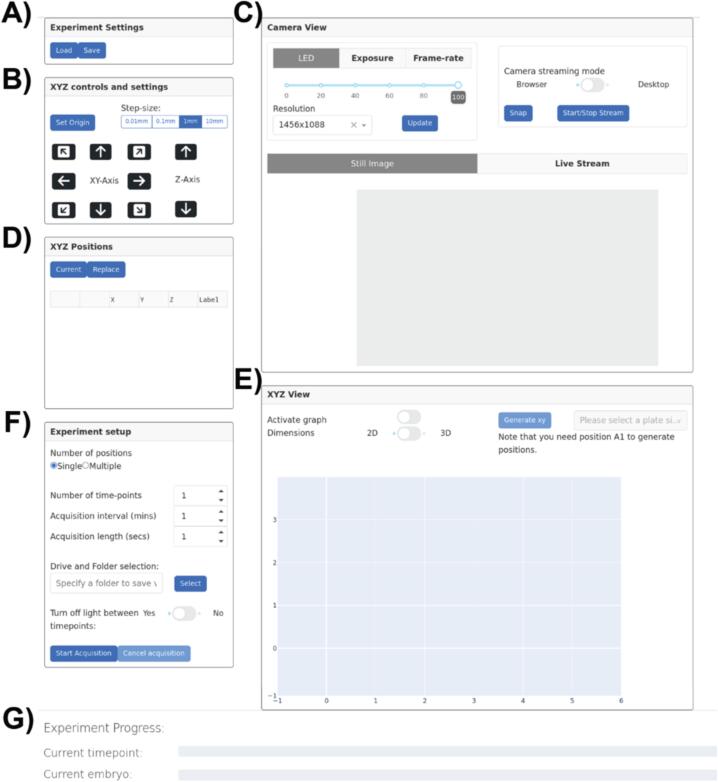
LabEmbryoCam User Interface. A) Experiment settings configuration and B) XYZ controls and settings, C) Camera live view and settings, D) XYZ position lists, E) XYZ position map, F) Experiment setup options, G) Live progress bars for the experiment.
• We can now proceed with getting the hardware set up for running an experiment. The first step required is to home the XYZ stage. This is essential to ensure that the correct origin is used when finding and creating positions. To do this, click the ‘Set Origin’ button in the ‘XYZ controls and settings’ section in the user interface (Fig. 12B) Before homing the stage, make sure there are no objects that could obstruct the movement of the stage.

• If using the provided disk image for the Raspberry Pi – the user icon will already be present on the Desktop of the Raspberry Pi.
• Otherwise, you can open the LEC user interface by navigating to the folder containing the LabEmbryoCam program and select ‘Open in Terminal’. Once the Terminal window is open, type: *python3* *app.py*
• Once you have started the LabEmbryoCam user interface you will be given an address to open in your browser and once opened you should see the user interface (Fig. 12).

### 6.3 Setting up an experiment

• Once the stage is homed (origin set), you can now make use of the manual controls to move the stage to desired positions (Fig. 12B). To record positions for an experiment, you need to first start a camera live stream to ensure your positions are correct. This can be achieved by clicking the ‘Start/Stop Stream’ button in the ‘Camera View’ section in the user interface (Fig. 12C)
• Now that you have a live stream from the camera, position the stage over a desired position by using the user interface controls and the live stream as feedback.
• Before changing any of the camera settings you must exit the live stream so that your changes can take effect. When you have finished choosing your desired settings, press the Update Settings button before starting the live stream again.
• Once you have a position that you want, click the ‘Current’ button in the ‘XYZ Positions’ section (Fig. 12). Note that you can either record your own positions, or save time by making use of the automated XYZ multiwell plate position generator.
  o To automatically generate positions for wells of a multiwell plate, first move to the centre of well ‘A1′ and click ‘Current’ – give this well the label ‘A1′. You can then choose a multiwell plate format from the dropdown at the top of the ‘XYZ View’ section – and click the ‘Generate xy’ (Fig. 12?).
  o If not all of the wells that are generated are required, you can delete these rows from the position list in ‘XYZ Positions’ (Fig. 12?).
  o You can also modify the positions using the manual controls to move the stage to a new position, and then selecting a position in the position list and clicking the ‘Replace’ button (Fig. 12?).
• To more easily visualise and navigate a multiwell plate, click the ‘Activate Graph’ toggle in the ‘XYZ View’ section (Fig. 12?). This will present you an interactive graph enabling you to move between positions by clicking on any point.
• Once all desired positions are listed in the XYZ position table, the next step is to enter the parameters for the acquisition before starting the experiment.These are located in the ‘Experiment Setup’ Section (Fig. 12)::
  o Number of positions − Whether you would like to capture footage for only the current position (‘Single’) or all the positions you have recorded (‘Multiple’).
  o Number of time-points − How many acquisition iterations you would like the system to complete. An iteration consists of recording video for each of the specified positions.
  o Acquisition interval − The time between each timepoint in minutes.
  o Acquisition length − How long to capture video for each position, at each timepoint.
  o Driver and folder selection − The full file path to the directory where you would like to save video. You can either specify this in the text input or click the button ‘Select’ which will open up the file explorer to interactively choose a folder.
  o Turn off light between timepoint − if enabled the illumination of the sample will be constant throughout the acquisition.
  o After entering the parameters for your experiment, the last step is to press the Start Acquisition button to begin the experiment. Note that you can cancel an acquisition in progress if you would like to make adjustments to the parameters used by the software.

### 6.4 Output of an experiment

• Individual AVI videos are saved for each time point within a folder labelled with the XYZ position label. Metadata on the acquisition and timings are also stored in capture_data.csv and time_data.csv (https://doi.org/10.5281/zenodo.7575249).
• Videos for each embryo can be compiled into a longer video covering the duration of the experiment using the script ‘compile_videos.py’ located in the analysis scripts folder of the LabEmbryoCam software.
• Either raw video from the LabEmbryoCam, or the compiled videos can be played using the ImageJ variant Fiji, or VLC media player. In the analysis_scripts folder within the LabEmbryoCam directory, there is a simple user interface for playing either individual videos via ‘timepoint_viewer.py’, or multiple timepoints via ‘multi-timepoint_viewer.py’, for a particular embryo.
• The video from the LabEmbryoCam can be used in a number of downstream analytical processes described in previously published research, including optical flow [5], heart rate detection [9], size, shape and movement measurement [7], or energy proxy traits [11].

## 7 Validation and characterization

### 7.1 Experiment setup and testing

To test and validate the design of the LabEmbryoCam an instrument was run for 48 h recording the development of nine early hippo stage embryos of the freshwater gastropod *Radix balthica*, generating forty eight 20 s videos. This testing, as well as substantial subsequent testing in our lab, demonstrated that the LEC is able to run unsupervised for extended periods (https://doi.org/10.5281/zenodo.7575249).

**Fig. 13:**
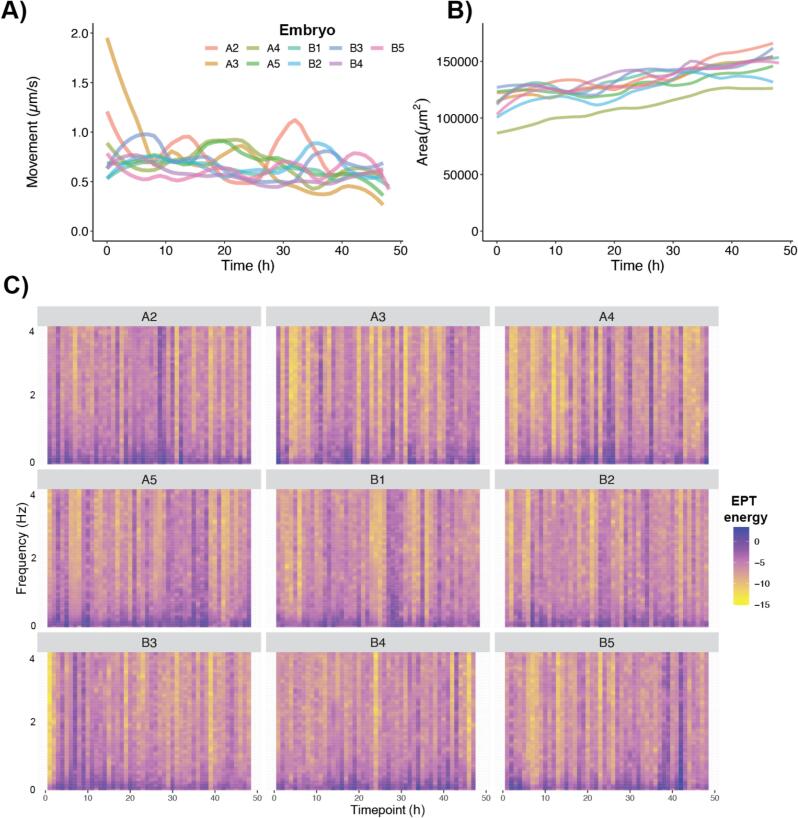
A) Developmental change in speed of the movement of individual embryos. B) Growth of individual embryos. C) Energy Proxy Trait Spectra (Tills et al 2021, 2023) for individual embryos during 48 h of development.

To assess the capability of video produced using the LabEmbryoCam for automated image analysis, it was analysed using the open-source Python package EmbryoCV [7]. EmbryoCV produced effective measures of change to size, movement, and also energy proxy traits – energy in the spectrum of fluctuating pixel values, an emerging transferrable phenotyping approach [8], [11]. The LabEmbryoCam was effective in generating video of a quality that enabled the extraction of continuous individual-level physiological data, including growth, movement and energy proxy traits (Fig. 13).

### 7.2 Humidification

The humidification chamber is an optional addition to the LabEmbryoCam to overcome the common evaporation issue associated with multiwell plates. Humidity > 90 % was achieved in the humidified airspace above a 96 multiwell plate (DHT22 humidity sensor) using a flow rate of 1 L/min for the duration of a 48 h test.

### 7.3 Camera and lens performance

**Fig. 14:**
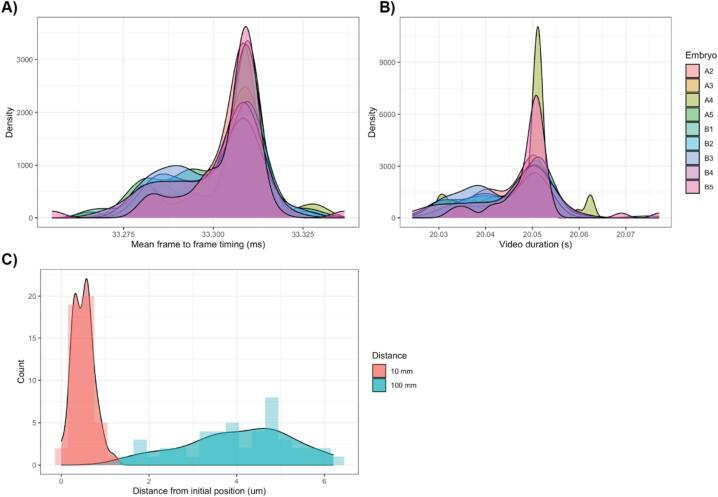
A) Density of mean frame to frame timings and B) video duration for nine embryos, imaged for 20 s at 30 fps, hourly during a 48 h period. C) Spatial repeatability of X and Y movements during two tests of 48 movements of 10 mm and 100 mm of the LabEmbryoCam in the X and Y axis directions.

The performance of the camera was assessed by examining the metadata from the 48 h camera to investigate consistency of frame to frame timings and video duration (Fig. 14). The camera was effective at operating at a range of resolutions, and corresponding upper frame rates (Table 2), and presets for these are included in the LabEmbryoCam web application.

**Table 2:** Frame rates for acquisition of video from the Raspberry Pi HQ camera, connected to a Raspberry Pi 4 via HDMI-CSI adapter (Arducam, Kowloon, Hong Kong) controlled using the LabEmbyoCam user interface.

**Resolution (pixels)**	**Lower frame rate(fps)** **> 10 ms exposure**	**Upper frame rate (fps)** **< 10 ms exposure**
640 x 480	30	90
1280 x 720	30	90
1920 x 1080	30	40
256 x 256	30	90
512 x 512	30	90
1024 x 1024	30	80
2048 x 2048	30	20

### 7.4 XYZ movement accuracy

With the aim of testing the accuracy of camera movements, two tests were carried out. We selected two positions to take 100 1 s videos per position every 5 min. A distance of 1 mm between positions was used for the first test and a distance of 100 mm used for the second (Fig. 14). To assess the accuracy of camera movements, images were taken of a calibration slide before and after movement. Using the open-source image analysis software ImageJ [12], distances moved (µm) were calculated and used to assess the accuracy of movement relative to the known distances.

### CRediT authorship contribution statement

**Ziad Ibbini:** Writing – review & editing, Writing – original draft, Visualization, Software, Investigation, Conceptualization. **Maria Bruning:** Writing – review & editing, Validation. **Sakina Allili:** Writing – review & editing, Validation. **Luke A Holmes:** Writing – review & editing, Validation. **Ellen Tully:** Writing – review & editing, Validation. **Jamie McCoy:** Writing – review & editing, Validation, Conceptualization. **Benjamin Larsen:** Visualization, Validation. **Tony Wilson:** Writing – review & editing, Validation. **Guy Ludford:** Writing – review & editing, Validation, Conceptualization. **Jack Barrett-Kelly:** Writing – review & editing, Validation, Conceptualization. **John I. Spicer:** Writing – review & editing, Conceptualization. **Oliver Tills:** Writing – review & editing, Writing – original draft, Visualization, Supervision, Project administration, Funding acquisition, Formal analysis, Conceptualization.

## Declaration of competing interest

The authors declare that they have no known competing financial interests or personal relationships that could have appeared to influence the work reported in this paper.
